# *KRAS* and *BRAF* Mutation Rates and Survival Outcomes in Colorectal Cancer in an Ethnically Diverse Patient Cohort

**DOI:** 10.3390/ijms242417509

**Published:** 2023-12-15

**Authors:** Paul Habashy, Vivienne Lea, Kate Wilkinson, Bin Wang, Xiao-Juan Wu, Tara Laurine Roberts, Weng Ng, Tristan Rutland, Joseph William Po, Therese Becker, Joseph Descallar, Mark Lee, Scott Mackenzie, Ruta Gupta, Wendy Cooper, Stephanie Lim, Wei Chua, Cheok Soon Lee

**Affiliations:** 1 Discipline of Pathology, School of Medicine, Western Sydney University, Sydney, NSW 2560, Australia; paul.habashy@gmail.com (P.H.); t.rutland@westernsydney.edu.au (T.R.);; 2 Liverpool Clinical School, Western Sydney University, Sydney, NSW 2170, Australia; tara.roberts@westernsydney.edu.au (T.L.R.); therese.becker@inghaminstitute.org.au (T.B.);; 3 Department of Anatomical Pathology, Liverpool Hospital, Sydney, NSW 2170, Australia; 4 Department of Medical Oncology, Liverpool Hospital, Sydney, NSW 2170, Australia; 5 Ingham Institute for Applied Medical Research, Liverpool Hospital, Sydney, NSW 2170, Australia; 6 South Western Sydney Clinical School, University of New South Wales, Sydney, NSW 2170, Australia; 7 Surgical Innovations Unit, Department of Surgery, Westmead Hospital, Sydney, NSW 2140, Australia; 8 Department of Radiation Oncology, Liverpool Hospital, Sydney, NSW 2170, Australia; 9 Department of Surgery, Liverpool Hospital, Sydney, NSW 2170, Australia; 10 Department of Tissue Pathology and Diagnostic Oncology, NSW Health Pathology, Royal Prince Alfred Hospital, Sydney, NSW 2050, Australia; 11 Sydney Medical School, University of Sydney, Sydney, NSW 2050, Australia; 12 Department of Medical Oncology, Campbelltown Hospital, Sydney, NSW 2560, Australia

**Keywords:** colorectal cancer, *BRAF*, *KRAS*, genetic screening, MMR, ethnically diverse, racial–ethnic

## Abstract

*KRAS* and *BRAF* mutation rates in colorectal cancer (CRC) reported from various mono-ethnic studies vary amongst different ethnic groups. However, these differences in mutation rates may not be statistically significant or may be due to differences in environmental and/or laboratory factors across countries rather than racial genetic differences. Here, we compare the *KRAS*/*BRAF* mutation rates and survival outcomes in CRC between ethnic groups at a single institution. We also investigate the contributions of genetic, environmental, and laboratory factors to the variations in *KRAS*/*BRAF* mutation rates reported from different countries. Clinicopathological data from 453 ethnically diverse patients with CRC were retrospectively analyzed at Liverpool Hospital, NSW Australia (2014–2016). *KRAS*/*BRAF* mutations were detected using real-time PCR (Therascreen kits from Qiagen). Mismatch repair (MMR) status was determined using immunohistochemical staining. Four ethnic groups were analyzed: Caucasian, Middle Eastern, Asian, and South American. Overall survival data were available for 406 patients. There was no significant difference in *KRAS* mutation rates between Caucasians (41.1%), Middle Easterners (47.9%), Asians (44.8%), and South Americans (25%) (*p* = 0.34). *BRAF* mutation rates differed significantly between races (*p* = 0.025), with Caucasians having the highest rates (13.5%) and Middle Easterners the lowest (0%). A secondary analysis in which Caucasians were divided into three subgroups showed that ethnic grouping correlated significantly with *KRAS* mutation rate (*p* = 0.009), with central and eastern Europeans having the highest rates (58.3%). There were no significant differences in overall survival (OS) or disease-free survival (DFS) between the four races. The similarity in *KRAS* mutation rates across races raises the possibility that the differences in *KRAS* mutation rates reported from various countries may either not be statistically significant or may be due to environmental and/or laboratory factors rather than underlying racial genetic differences. In contrast, we verified that *BRAF* mutation rates differ significantly between races, suggesting racial genetic differences may be responsible for the discrepant *BRAF* mutation rates reported from different countries.

## 1. Introduction

Colorectal cancer (CRC) is the third most common cancer diagnosed globally [1]. *KRAS* and *BRAF* mutations play an important role in the pathogenesis of CRC. KRAS is a small protein possessing GTPase activity that lies downstream of the epidermal growth factor receptor (EGFR) and activates the BRAF/MAPK and PI3K/Akt pathways in cell signaling, which promote cell survival and proliferation. Activating mutations in *KRAS* occur in 35–45% of CRC cases [2,3] BRAF is a serine-threonine protein kinase downstream of RAS. The majority of *BRAF* mutations in sporadic colorectal tumors occur in tumors with microsatellite instability (MSI) [4].

The current treatment strategy for stage IV CRC includes the use of EGFR monoclonal antibodies (mAbs) in *KRAS* wild-type (*KRAS*-WT) tumors to suppress the EGFR-RAS signaling pathway that drives cellular survival and proliferation. Determining the tumor *KRAS* status in a patient with stage IV CRC is important, because *KRAS*-mutant CRC is resistant to EGFR monoclonal antibodies (mAbs) as the mutant RAS continues the activation of the MAPK pathway downstream of EGFR-RAS.

The majority of *KRAS* mutations in CRC occur in codons 12 and 13. The common ones include G12A, G12C, G12D, G12V, and G13D, and less common ones include G12S, G12R, and G13A. Specific mutations have been associated with certain clinicopathological features. For example, G12A, G12C, and G12V mutations are associated with a poorer prognosis, while tumors with G13D mutations, unlike tumors with other KRAS mutations, are sensitive to EGFR inhibition [5].

The *KRAS* and *BRAF* mutation rates reported in the literature vary according to the study’s country, suggesting that *KRAS* and *BRAF* mutation rates may differ between races [6,7]. KRAS mutation rates vary widely among Asian studies (13–66.1%) [8,9,10,11,12,13,14,15,16,17,18,19,20], Western studies (12.9–53.8%) [11,21,22,23], Middle Eastern studies (11–56%) [24,25,26,27,28,29,30,31], and South American studies (43.3–59%) [32,33,34]. Higher *KRAS* mutation rates have been reported among African Americans compared to Caucasians [35], while a review showed that Hispanics had a higher rate of *KRAS* mutations than Caucasians (though this was not statistically significant) [11]. The *BRAF* mutation rates reported in Asian studies tend to be lower than those reported in Western studies [11,12,14,17,19,20,36,37,38,39,40,41,42]. However, it is difficult to determine whether these differences in *KRAS* and *BRAF* mutation rates reported from independent studies represent true racial differences, because no statistical tests were performed to compare the studies’ cohorts and the mutation detection methods may have varied between studies. In this study, we recruited patients from various races into a single study and performed statistical tests to compare the *KRAS* and *BRAF* mutation rates across races.

If the differences in *KRAS* and *BRAF* mutation rates reported from various countries are indeed statistically significant, then there are three possible explanations to account for these reported differences: genetic differences between races; the influence of environmental factors, such as diet, that differ across countries; and heterogeneity in the laboratory techniques used across studies, such as the choice of KRAS codons analyzed. In this study, we recruited an ethnically diverse patient cohort from the same geographical location, whose tissue specimens were subjected to the same laboratory analyses. This helps to eliminate heterogeneity in environmental factors and laboratory techniques and allows us to better elucidate the contributions of racial genetic differences and environmental and laboratory factors to the discrepancies in the *KRAS* and *BRAF* mutation rates reported from different countries.

Survival outcomes for CRC have been reported for different races in several mono-ethnic studies, but comparing the survival outcomes between ethnic groups using these studies may be invalid due to heterogeneity in study populations and an inability to control for potential confounders such as the disease stage, age of patients, and receipt of chemotherapy. Survival outcomes have been validly compared between Caucasians and African Americans by recruiting participants simultaneously from a single institution [43], but this has not been frequently performed for other races. In this study, we recruited patients from a single institution and thus are able to control for potential confounders when investigating racial differences in survival outcomes.

South Western Sydney Local Health District (SWSLHD) services a geographical area with an ethnically diverse patient population, providing us a unique opportunity to investigate the racial differences in *KRAS* and *BRAF* mutation rates and survival outcomes in CRC. It also allows us to elucidate the contributions of genetic, environmental, and laboratory factors to the differences in the *KRAS* and *BRAF* mutation rates reported from various countries.

## 2. Results

### 2.1. Patient Characteristics and Clinicopathological Variables

In total, 453 patients were included in the study. A total of 48.6% of patients presented with stage IV disease. Of the 453 patients, 294 (68.1%) were Caucasian, 50 (11.6%) were Middle Eastern, 68 (15.7%) were Asian, and 20 (4.6%) were South American. The median age at diagnosis was 66 years. Age at diagnosis differed significantly between the four races (*p* < 0.001), with Asians being diagnosed at the youngest age. Excepting *BRAF* status and age, there were no significant associations between race and any of the clinicopathological variables (Table 1).

### 2.2. Tumor Characterisitcs

**Figure 1:**
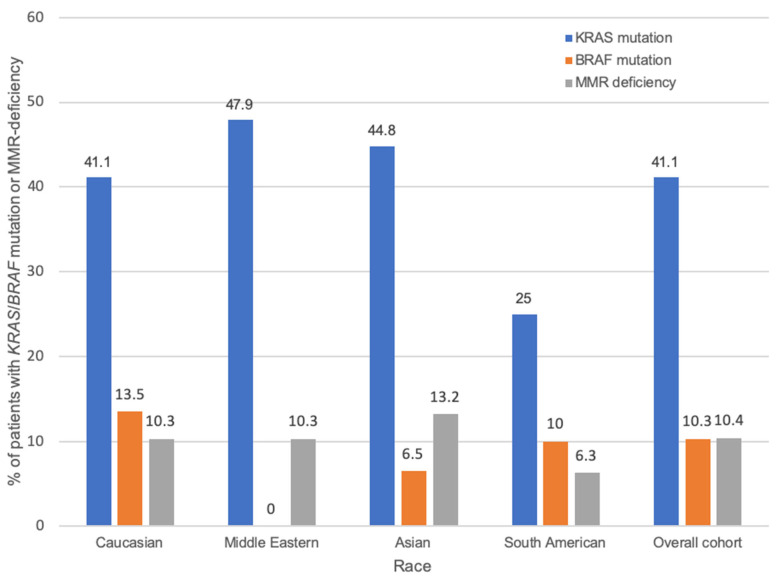
Frequency of *KRAS* and *BRAF* mutations and MMR deficiency within each race and in the overall cohort.

*KRAS* and *BRAF* were mutated in 41.1% and 10.3% of patients, respectively (Figure 1). MMR-deficiency was present in 10.4% of patients. *KRAS* mutation was associated with MMR proficiency and *BRAF* wild-type (*BRAF*-WT). Only 5/387 tumors were both *KRAS*-mutant and *BRAF*-mutant (one G12R, two G12V, and two G12D *KRAS* mutations). Only 6/328 tumors were both MMR-deficient and *KRAS*-mutant. *KRAS*-WT was associated with poor differentiation, vascular invasion, and circumferential tumors. *BRAF* mutation was associated with MMR deficiency, poor differentiation, and right-sided tumors. MMR deficiency was associated with older age, poor differentiation, the presence of tumor-infiltrating lymphocytes, and right-sided tumors. MMR-proficient tumors were associated with a higher N-stage and perineural invasion (Table 2).

### 2.3. Race and KRAS/BRAF

There was no significant association between race and *KRAS* status in the overall cohort (Table 3), and this remained unchanged after stratification by MMR status and tumor location (Tables S1 and S2). Among patients with right-sided tumors, Asians had a higher frequency of *KRAS* mutations, but this did not reach statistical significance (*p* = 0.055).

There was a significant association between *BRAF* mutation frequency and race (*p* = 0.025), with 0% of Middle Easterners having a *BRAF* mutation and Caucasians having the highest rate (13.5%) (Table 3). This association between race and *BRAF* status was confined to MMR-deficient tumors (Table S1), but the small number of MMR-deficient tumors necessitates caution in interpreting this subgroup analysis.

The frequencies of specific *KRAS* mutations in our cohort are shown in Supplementary Table S3. For each specific mutation tested (G12C, G12R, G12S, G12A, G12D, G12V, and G13D), there was no significant difference between races for the frequency of that mutation (Table 4).

Only one patient in our cohort had a non-V600E *BRAF* mutation. This was a Caucasian with a V600R mutation.

### 2.4. Survival Outcomes

#### 2.4.1. Race

**Figure 2:**
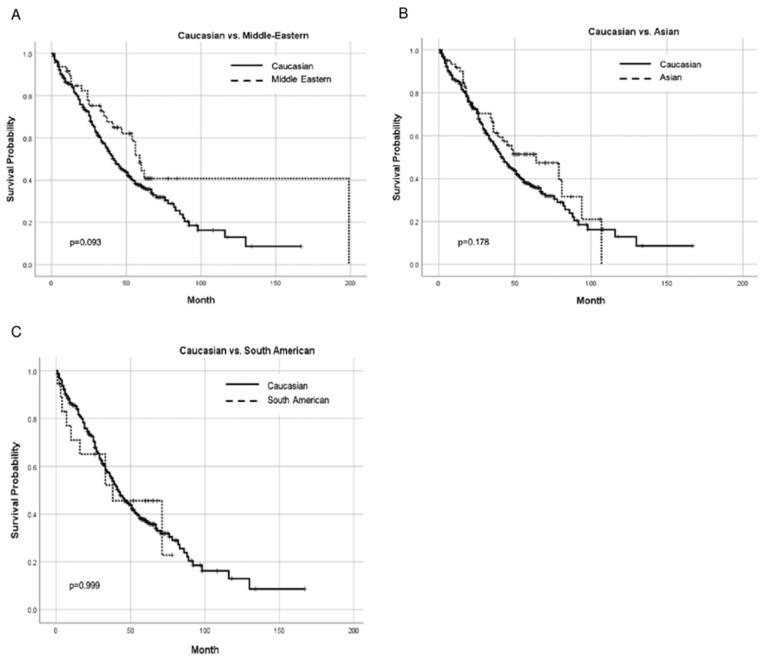
Kaplan–Meier curves comparing OS between Caucasians and other races: (**A**) Caucasian vs. Middle Eastern; (**B**) Caucasian vs. Asian; and (**C**) Caucasian vs. South American. Median follow-up time in months: Caucasian 64, Middle Eastern 64, Asian 62, and South American 62.

Of the 453 patients, data for overall survival (OS) were available for 406 patients. There was no difference in OS between any pair of races (Figure 2), and this was unchanged after stratification by *KRAS* status, MMR status, and tumor location.

We attempted to compare the OS between races after grouping the patients by their specific *KRAS* mutation (G12D/G12R/G12V/G12A/G12C/G12S/G13D). Among the patients with *KRAS* G12D mutations (the most abundant mutation in our cohort), there was no significant difference in OS between Caucasians and Middle Easterners (median OS: 39 vs. 41 months, *p* = 0.93). There was no difference in OS between Caucasians and Asians for G12V (21 vs. 36 months, *p* = 0.6) and G12A (21 vs. 36 months, *p* = 0.8) mutations. However, these results must be interpreted with caution due to the small number of patients in the non-Caucasian subgroups. The relevant Kaplan–Meier curves are shown in Supplementary Figure S1.

With respect to the other *KRAS* mutations and other racial groups, there were too few patients remaining in each racial subgroup after stratification by mutation type to allow for a meaningful survival analysis.

**Figure 3:**
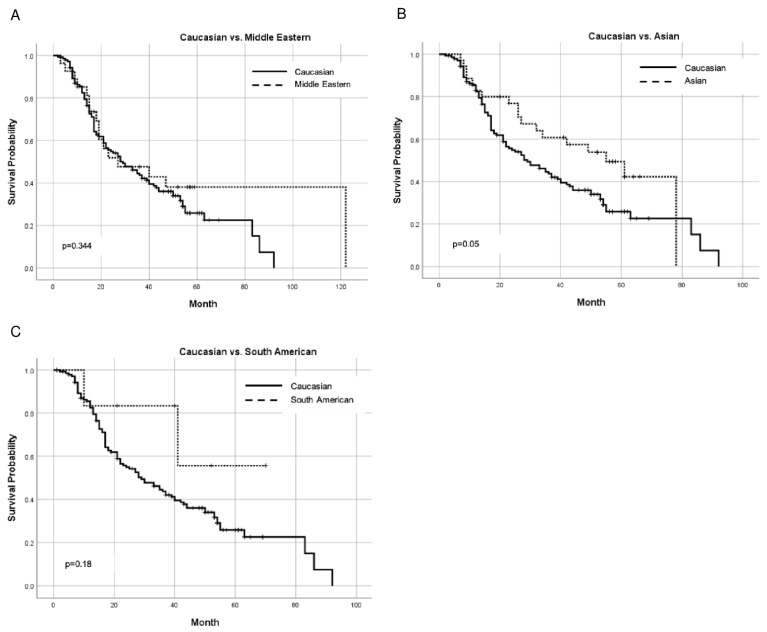
Kaplan–Meier curves comparing DFS between Caucasians and other races: (**A**) Caucasian vs. Middle Eastern; (**B**) Caucasian vs. Asian; and (**C**) Caucasian vs. South American. Median follow-up time in months: Caucasian 54, Middle Eastern 56, Asian 57, and South American 40.

Out of the 453 patients in the study, DFS could be calculated for 216 patients who achieved complete remission after treatment and for whom follow-up data were available. Asians had a longer DFS than Caucasians (median DFS: 55 vs. 29 months), but the difference in survival curves did not reach statistical significance (*p* = 0.05) (Figure 3). There were no significant differences in DFS for all other pairs of races.

**Figure 4:**
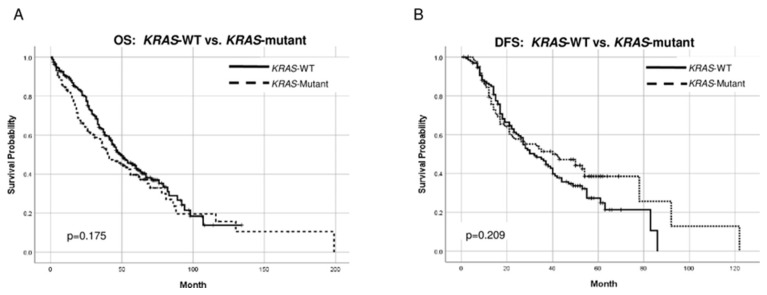
Kaplan–Meier curves comparing OS and DFS between patients with *KRAS*-WT and *KRAS*-mutant tumors: (**A**) OS; (**B**) DFS. Median follow-up time in months (OS analysis): *KRAS*-WT 62, *KRAS*-mutant 62. Median follow-up time in months (DFS analysis): *KRAS*-WT 54, KRAS-mutant 56.

The median follow up times, as calculated using the reverse Kaplan–Meier method, are reported in Figure 2, Figure 3 and Figure 4.

#### 2.4.2. *KRAS*/*BRAF*/MMR Status

Although patients with *KRAS*-WT tumors had a better OS than patients with *KRAS*-mutant tumors (49 vs. 40 months), the difference in survival curves did not reach statistical significance in the overall cohort (*p* = 0.175) or when stratified by location, MMR status, *BRAF* status, and race (Figure 4).

For each race, we attempted to analyze the effect of the type of *KRAS* mutation on OS. There was no difference in the OS between patients with G12D mutations and patients without G12D mutations among the Caucasian (39 vs. 42 months, *p* = 0.63) and Middle Eastern (41 vs. 56 months, *p* = 0.69) groups. There were insufficient sample sizes to perform valid subgroup analyses for the other *KRAS* mutations or other racial groups. The relevant Kaplan–Meier curves are shown in Supplementary Figure S1.

*BRAF* mutation was not associated with worse OS in the overall cohort or the MMR-deficient subgroup, but was associated with a worse OS in the MMR-proficient subgroup (*p* = 0.011). However, the sample sizes were too small to properly compare the OS between patients with *BRAF*-WT and *BRAF*-mutant tumors.

Neither *KRAS* nor *BRAF* status impacted DFS.

Among the patients with stage IV CRC, patients with *KRAS*-mutant CRC had a worse OS than patients with *KRAS*-WT CRC, though the result did not reach statistical significance (20 vs. 34 months, *p* = 0.10). Among the subgroup of Caucasians with stage IV CRC, *KRAS*-mutant CRC was associated with a worse OS than *KRAS*-WT CRC (19 vs. 33 months), with the difference approaching statistical significance (*p* = 0.053). *KRAS* status did not impact OS in stage IV CRC among Middle Easterners (*p* = 0.18) or Asians (*p* = 0.93).

There was no difference in the OS between *KRAS*-WT and *KRAS*-mutant tumors among patients with stage I-III CRC (76 vs. 78 months, *p* = 0.90), and this was unchanged after stratification by race.

The relevant Kaplan–Meier curves are shown in Supplementary Figure S2.

There was no significant difference in the OS between *BRAF*-WT and *BRAF*-mutant tumors among patients with stage IV CRC (*p* = 0.137), or among patients with stage I-III CRC (*p* = 0.71).

#### 2.4.3. Multivariable Analysis

A multivariable Cox model including known prognostic factors in CRC was constructed involving 203 patients (Table 5). The variables that were predictive of OS in the multivariable Cox model, at a cut-off of *p* < 0.05, were age, *BRAF* status, MMR status, AJCC stage, tumor differentiation, and receipt of chemotherapy. In particular, race was not predictive of OS, and there was no interaction between race and *KRAS* status. The full details of the Cox model are provided in Supplementary Table S4.

### 2.5. Six Ethnic Groups

Although our primary analysis utilized four races, we subsequently split Caucasians into Anglo-Saxons, Southern Europeans, and Other Europeans, and conducted secondary analyses using a total of six ethnic groups (Table 6). There was a significant association between ethnic group and *KRAS* status (*p* = 0.009), with Other Europeans having a significantly higher mutation rate than other ethnic groups. *BRAF* status continued to be associated with ethnic group (*p* = 0.01), and this was again confined to MMR-deficient tumors, in accordance with our primary analysis using four races.

There was no significant difference in OS between the six ethnic groups in the overall cohort. However, among MMR-proficient tumors, Anglo-Saxons had a worse OS than Middle Easterners (*p* = 0.037). Among *KRAS*-WT tumors, Anglo-Saxons had a worse OS than Other Europeans (*p* = 0.043). In the overall cohort, Anglo-Saxons had a worse DFS compared to Asians (*p* = 0.006) and Other Europeans (*p* = 0.03). Left-sided tumors were associated with a better OS in only Anglo-Saxons (*p* = 0.018). *KRAS* status was not prognostic among any of the six ethnic groups. Otherwise, all other analyses yielded similar results to those derived from the primary analysis utilizing four races.

## 3. Discussion

We conducted a cross-sectional study of the *KRAS* and *BRAF* mutational landscape of patients with CRC, whose tumor specimens were analyzed at a single institution. We compared the *KRAS* and *BRAF* mutation rates and OS and DFS between four races in a primary analysis, then between six ethnic groups in a secondary analysis after splitting Caucasians into three European subgroups. To our knowledge, this is the first study that has compared four or more ethnic groups from a single institution.

The *KRAS* and *BRAF* mutation rates in the overall cohort were 41.1% and 10.3%, respectively, which are similar to the rates reported in the literature. The associations we found between *KRAS* status and vascular invasion, differentiation, and circumferential tumors are consistent with prior studies [44,45]. Likewise, the associations between *BRAF* mutation and poor differentiation, MMR deficiency, and right-sided tumors have been reported [46]. We also verified the mutual exclusivity between *KRAS* and *BRAF* mutations [5].

For many patients with stage IV CRC, we determined the *KRAS*/*BRAF* status of an accessible metastatic lesion and assumed it was the same as that of the primary tumor. This technique is valid, as studies have shown a high concordance rate for KRAS/BRAF mutations between primary and metastatic lesions [47,48].

We showed that there were no significant racial differences in the *KRAS* mutation rates between Caucasians, Middle Easterners, Asians, and South Americans, but that the *BRAF* mutation rates differed significantly between these races. *BRAF* mutations were most frequent among Caucasians (13.5%), consistent with a systematic review comparing Caucasians, African Americans, and Asians [49], and least common among Middle Easterners (0%). The low prevalence of *BRAF* mutations in Middle Easterners is supported by two large studies in Saudi Arabia [36] and Iran [28], which reported mutation rates of 2.5% and 0%, respectively.

The *KRAS* and *BRAF* mutation rates reported from various mono-ethnic studies have varied, but it is unclear whether these differences are statistically significant, and if so, whether they are due to racial genetic differences or to environmental/laboratory factors that differ between countries. Environmental factors such as diet may lead to gene mutations through the effect of carcinogens. For example, low folate intake has been associated with *KRAS* mutations in CRC [50], while dietary fibre has been associated with a reduced risk of *KRAS* mutations [51]. Variations in laboratory techniques across different studies may also account for the discrepant *KRAS* mutation rates reported from various countries. We have shown in this study that, in a cohort of patients residing in the same region and whose tumor specimens were analyzed in the same laboratory, there is likely no racial difference in *KRAS* mutation frequency, but there is likely a significant racial difference in *BRAF* mutation frequency. This raises the possibility that the differences in KRAS mutation rates reported from various countries may not be statistically significant, or may be due to differences in environmental or laboratory factors across countries rather than underlying racial genetic differences. On the other hand, underlying racial genetic factors may contribute more heavily to the discrepant *BRAF* mutation rates reported in the literature. However, this suggestion should be taken cautiously, as we could not confirm that environmental factors such as diet were uniform across our cohort.

The similarity of *KRAS* mutation rates across races may suggest that the chromosomal instability (CIN) pathway occurs at similar rates across races, whereas the difference in *BRAF* mutation rates between races may suggest that the sporadic MSI pathway and the CpG Island Methylator Phenotype (CIMP) pathway occur more frequently in Caucasians and less frequently in Middle Easterners.

An interesting result is that none of the 39 Middle Eastern patients had a *BRAF* mutation. This may suggest that *BRAF* mutations have a smaller role in the pathogenesis of CRC in Middle Easterners than in other races. Since *BRAF* mutations occur almost exclusively in CIMP-high tumors with MSI [52], our results suggest that the sporadic MSI and CIMP pathways may be less common among Middle Easterners. *BRAF* mutations occur early in the process of CRC tumorigenesis, and cancer progression in *BRAF*-mutant pre-cancerous lesions is unlikely to occur in the absence of other factors such as CIMP [53]. Middle Easterners have been shown to have a low frequency of CIMP, with a frequency of 4.8% reported in Saudi Arabia [36,53]. Thus, the low frequency of *BRAF* mutations we observed among Middle Easterners may reflect a primary predilection among this racial group towards *BRAF*-WT lesions, or may instead simply reflect the low frequency of CIMP in Middle Easterners that is essential for the progression to cancer of BRAF-mutant lesions.

*BRAF* mutations are rare in familial syndromes of CRC. This raises the question of the prevalence of familial CRC syndromes such as Lynch syndrome among Middle Easterners. Current Australian guidelines [54] recommend genetic testing for Lynch syndrome in all patients whose tumors are both MMR-deficient and *BRAF*-WT, because these patients have a higher risk of germline mutations in the MMR genes. Our data showed that, although *BRAF* mutations were significantly less frequent among Middle Easterners, MMR deficiency occurred at similar rates across all races. This may have implications for the burden of Lynch syndrome and Lynch syndrome screening practices among Middle Easterners. Indeed, a Saudi Arabian study [55] showed that, out of 33 cases of tumors with MSI, only one had a *BRAF* mutation. In light of the low rate of *BRAF* mutations among Middle Easterners, the current recommended algorithm for Lynch syndrome screening in Western countries may be less cost-effective in Middle Eastern populations, in which immediate recourse to germline testing following a demonstration of MMR deficiency may be more cost-efficient than first testing for *BRAF* mutations.

With respect to survival outcomes, we showed that there were no racial differences in OS. In contrast, many US studies have reported survival differences between Caucasians and African Americans [43,56]. While this disagreement between our results and these US studies may be due to the absence of African Americans in our cohort, it may also reflect a fundamental difference between the US and Australian health systems. A larger proportion of African Americans are socioeconomically disadvantaged compared to Caucasians, and this is one potential contributor to their poorer survival [57]. The provision of Medicare (universal healthcare) in the Australian healthcare system may tend to blunt the impact of socioeconomic status on disease outcomes that may apply in the US. Thus, the racial discrepancy in survival outcomes reported in US studies may be due to socioeconomic factors rather than racial differences in cancer biology. This corroborates the results of a large study involving 131,481 women with CRC, in which the authors suggest that socioeconomic factors rather than race were the main cause for the discrepant survival outcomes between Caucasians and African Americans [58]. Another study showed that, among patients with stage II and III rectal cancer, socioeconomic status completely accounted for the discrepancy in survival between Caucasians and African Americans [59].

Studies have shown conflicting results about the impact of *KRAS* status on survival in CRC. One systematic review and meta-analysis did not show any association between *KRAS* status and OS in patients receiving chemotherapy [60]. Other studies have shown that *KRAS* mutations confer a worse prognosis [31]. Indeed, stage IV *KRAS*-mutant CRC is not amendable to anti-EGFR therapy, so patients with stage IV *KRAS*-mutant CRC may have worse outcomes [61]. In our overall cohort, patients with *KRAS*-mutant tumors had a worse OS than patients with *KRAS*-WT tumors, but this was not statistically significant (*p* = 0.175), even after stratification by race. Moreover, in the multivariable Cox model, there was no interaction between race and *KRAS* status. Among stage IV CRC, *KRAS*-mutant CRC was associated with a worse OS than *KRAS*-WT CRC (20 vs. 34 months), but the result was not significant (*p* = 0.1). However, in the subgroup of Caucasians with stage IV CRC, OS was worse in patients with *KRAS*-mutant CRC compared to *KRAS*-WT CRC, with the difference approaching statistical significance (*p* = 0.053). We were unable to analyze the effect of specific *KRAS* mutations on survival due to insufficient sample sizes. This may explain why we did not detect a difference in survival outcomes between *KRAS*-WT and *KRAS*-mutant CRC, as different mutations are associated with different survival outcomes [62]. Moreover, we did not account for patient comorbidities when assessing survival outcomes, which may also explain why we failed to detect a difference in OS between *KRAS*-WT and *KRAS*-mutant CRC.

Our study did not show that *BRAF* mutations were associated with a worse OS in the overall cohort, which differs from previous studies. This may be due to the small number of *BRAF*-mutant tumors in our cohort, limiting the statistical power. However, we did show that *BRAF* mutations were associated with a worse OS in the MMR-proficient subgroup (*p* = 0.011), which is consistent with previous studies [63].

There is some evidence in the literature of genetic heterogeneity among Europeans. For example, a large meta-analysis reported the highest *KRAS* mutation rates in CRC in Poland and the Czech Republic, both central European countries [3]. Seldin et al. showed, using a genome-wide single nucleotide polymorphism panel, consistent differences between northern and southern European groups [64]. We therefore conducted additional analyses after splitting the Caucasian group into Anglo-Saxon, Southern European, and Other European subgroups, and found that Other Europeans had a significantly higher *KRAS* mutation rate, which is consistent with the aforementioned meta-analysis [3]. Studies on CRC that include race often group all Europeans into a single ‘Caucasian’ cohort. Our data suggest that Europeans may represent a heterogeneous cohort and that pathways of CRC development may differ between Anglo-Saxons and central/eastern Europeans. A smaller proportion of central and eastern Europeans may benefit from anti-EGFR therapy compared to other races, and this may have implications for public health policies in those countries. In our local multiethnic health district, this information may have clinical implications for the types of treatment patients receive, including anti-EGFR mAbs, and for the design of clinical trials targeting specific *KRAS* mutations.

The main strength of this study is the racial diversity of patients treated at a single institution. Recruiting patients from a single institution and using the same laboratory to conduct genomic analyses on tissue specimens reduces heterogeneity in the management of patients and in the genomic analysis of patient samples.

The absence of patients from an African background in this study is a major limitation. African Americans have been shown to have higher *KRAS* mutation rates than Caucasians [35]. Therefore, any correlations drawn in this study between race and a given variable may only be applicable to the specific four races analyzed in this study. In particular, our suggestion that *KRAS* mutation rate is independent of race may only be applicable to the four races here examined.

Second, the small number of patients comprising the non-Caucasian groups limits the statistical power. The number of South Americans was especially small (20 patients), increasing the possibility of a type II error. Moreover, the large proportion of Caucasians in our cohort (comprising 68.1% of our patients) necessarily results in an unbalanced distribution in the Kaplan–Meier analyses. The numbers of patients in the MMR-deficient and *BRAF*-mutant subgroups were small, but this was expected. Third, pairwise exclusion was utilized to deal with patients with missing data, which can introduce bias. Fourth, we have argued that recruiting patients from the same geographical location reduces the impact of environmental factors on *KRAS* and *BRAF* status. However, this assumption may not be true, because environmental factors such as diet may still differ across ethnic groups living in the same location; moreover, patients who migrated to Australia more recently may still be experiencing lingering effects of their native country’s environment to a greater extent than patients who migrated a long time ago. Fifth, expanded *RAS* testing, which is now standard of care, was not readily available at the time our patients were referred for mutational testing. Sixth, we were unable to robustly compare survival outcomes between races for each specific type of *KRAS* mutation, as the small number of patients remaining in each racial group after stratification by type of *KRAS* mutation made analysis difficult. Similarly, all the patients in our cohort that had a *BRAF* mutation had the V600E mutation, except one patient who had a V600R mutation. This prevented us from analyzing racial differences in non-V600E *BRAF*-mutant tumors. Finally, ethnicity was determined using a combination of country-of-birth and surname. A more accurate method would have been to use self-reported ethnicity or a genetic ancestry analysis, but these were not available. We also included all south Asians, east Asians, and southeast Asians in our Asian group. Further studies should examine the differences between these Asian subgroups.

Finally, we compared our results to publicly available data from the AACR Project GENIE v. 11.0 cohort [65]. *KRAS* was mutated in 42% (3517/8315) of Caucasians, 41% (257/622) of Asians, and 47% of Hispanics (403/853). *BRAF* was mutated in 11% (943/8315) of Caucasians, 8% (49/622) of Asians, and 9% (77/853) of Hispanics. We performed Chi-square tests to check for associations between ethnic group and *KRAS*/*BRAF* status in the GENIE cohort. Our analysis showed that there was no significant difference in the *KRAS* mutation rate between Caucasians and Asians, but there was a correlation between ethnic group and *KRAS* status when the three ethnic groups were used (Chi-square = 8.24, *p* = 0.016), with Hispanics having a significantly higher *KRAS* mutation rate. There was a significant correlation between race and *BRAF* status when the three ethnic groups were used (Chi-square = 10.58, *p* = 0.005), with Asians having a significantly lower *BRAF* mutation rate. The similarity in *KRAS* mutation rates between Caucasians and Asians in the GENIE cohort, and the lower *BRAF* mutation rate among Asians compared to Caucasians, both agree with our results. However, our *KRAS* mutation rate for South Americans was lower than the rate reported for Hispanics in the GENIE cohort; this may have been due to the small number of South Americans in our study, or to the broader group that the term ‘Hispanic’ encompasses compared to the term ‘South American’. Middle Easterners were not represented as a separate group in the GENIE cohort, so we could not directly verify our results for Middle Easterners.

## 4. Materials and Methods

### 4.1. Study Population and Clinicopathological Data

Institutional ethics approval for this study was obtained from the Human Research Ethics Committee of the South Western Sydney Local Health District (HREC/12/LPOOL/102). The institutional review board waived the need for written informed consent from the participants, as the project was deemed to be in the low-risk category. Information was de-identified prior to analysis. The study population consisted of patients diagnosed with CRC in the years 2014–2016 and whose tumor specimens underwent genomic testing at Liverpool Hospital, NSW, Australia. Data were obtained from South Western Sydney Local Health District (SWSLHD) electronic medical records (eMR), NSW Health Laboratory Information System, and the SWSLHD Cancer Registry (known as Mosaiq). Most patients had a colonic resection. For each patient, regardless of whether they underwent a resection, the following clinicopathological information was recorded: presence and type of *KRAS* and *BRAF* mutations; MSI status; age; sex; ethnicity; AJCC stage at presentation; location of primary tumor; histological subtype of tumor; and treatments received, including neoadjuvant, adjuvant, or palliative chemotherapy. Additional information collected for patients who underwent colorectal resection included the presence of vascular, perineural, and lymphatic invasion, T stage, N stage, number of nodes involved, tumor differentiation, presence of tumor-infiltrating lymphocytes, presence of circumferential tumor, and maximum size of tumor.

Location was classified into right colon (caecum to splenic flexure, excluding splenic flexure) and left colon (splenic flexure to rectum, inclusive). The AJCC 8th edition was used for staging. Ethnicity was determined by a combination of both country-of-birth and surname, ensuring that non-Caucasians born in Australia were not labelled Caucasian. Patients from Maori/Islander or African backgrounds were excluded due to the small number of cases, resulting in an inadequate statistical power for analysis. We used four ethnic groups in the primary analysis: Caucasian, Middle Eastern, Asian, and South American. The Asian group consisted of east Asians, south Asians, and southeast Asians. Finally, given there is some evidence in the literature of genetic heterogeneity amongst Europeans [64], we then split Caucasians into three subgroups (Anglo-Saxon, Southern European, and Other European) and conducted a secondary analysis using a total of six ethnic groups.

### 4.2. Survival Outcomes

For each patient, the date of diagnosis and date of last follow up were recorded, the former being defined as the date on which CRC was diagnosed by histopathology (biopsy or resection). The primary endpoints were death and, where applicable, disease recurrence. Overall survival (OS) was defined as the time from diagnosis to death from any cause. Patients who did not die were censored at the date of last follow-up. Disease-free survival (DFS) for patients who achieved complete remission after treatment was defined as the time from diagnosis of curative disease to first radiological or histological evidence of recurrence, or if no recurrence, to the date of last follow-up. For six patients, the exact date of the scan showing recurrence was not available in the records; in these patients, the date/month of recurrence was estimated from the entries made by staff in the patient records.

The median follow up times were calculated using the reverse Kaplan–Meier method.

### 4.3. Somatic Mutation Analysis

The presence and type of *KRAS* and *BRAF* mutations and MSI status were determined for each patient’s tumor sample. For patients who underwent colonic resection, an analysis was performed on the primary tumors; for patients who presented with metastatic disease and did not undergo colonic resection, an analysis was performed on biopsied tissue samples from the primary or metastatic sites.

*KRAS* mutation detection was performed using real-time PCR and the *KRAS* RGQ PCRTherascreen kit (from Qiagen in Clayton, Melbourne, Australia), which detects the 7 mutations in codons 12 and 13 of the *KRAS* gene: G12C (c.34G>T), G12R (c.34G>C), G12S (c.34G>A), G12A (c.35G>C), G12D (c.35G>A), G12V (c.35G>T), and G13D (c.38G>A). *BRAF* mutation was detected using the Qiagen *BRAF* V600E RGQ PCR kit (from Qiagen in Clayton, Melbourne, Australia), which detects the following mutations in the *BRAF* gene: V600E, V600E complex, V600D, V600K, and V600R.

### 4.4. Determination of MMR Status

MMR status was determined via immunohistochemical staining for the MMR proteins MLH1, MSH2, MSH6, and PMS2. Tumors were classified as MMR-deficient if they lacked one or more of these proteins, and MMR-proficient if all four proteins were present on staining.

MMR testing was performed on the patients’ tumor samples as part of their routine diagnostic workup at the time of disease diagnosis. The stain results were interpreted by qualified pathologists. If staining for a particular MMR protein was positive, then that protein was deemed to be present in the tumor sample; if staining was negative, then the tumor was deemed to be deficient for that protein. If staining was equivocal/unclear, routine laboratory practice was to repeat the staining. None of the pathologists involved in interpreting the stains were involved in this study, as the staining was performed during the routine diagnostic workup of the patients, years before the current study was conceived.

**Figure 5:**
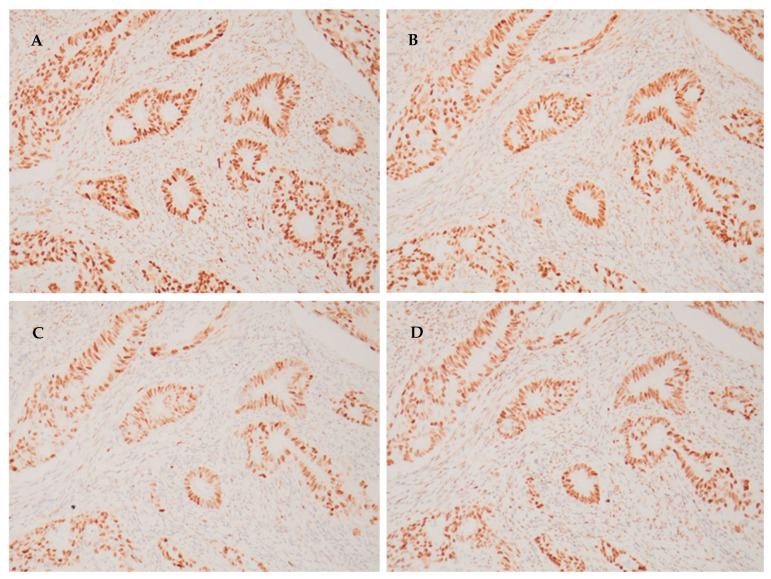
An example of positive immunohistochemical staining for each of the four MMR proteins (×200 magnification): (**A**) MLH1 positive; (**B**) MSH2 positive; (**C**) MSH6 positive; and (**D**) PMS2 positive.

As an example of positive staining, Figure 5 shows a tumor sample from one of the patients in our cohort that stained positive for all four proteins (MLH1, MSH2, MSH6 and PMS2), and was thus deemed to be MMR-proficient.

### 4.5. Statistical Analyses

Correlations between categorical variables were investigated using Fisher’s exact test. Before comparing age at diagnosis across two or more groups, the Shapiro–Wilk test was used to check for normality in the age distribution within each group, which showed non-normal distributions in several groups. A comparison of age across two groups or across more than two groups where age had a non-normal distribution in at least one group was performed using the Mann–Whitney U and Kruskal–Wallis tests, respectively. For each analysis, all patients with available data for the relevant variables were included, and patients missing data for one or more of the relevant variables were excluded (i.e., pairwise exclusion).

Racial differences in OS and DFS were investigated by constructing Kaplan–Meier curves for each race and comparing the survival curves pairwise using the log-rank test. This was performed with the entire cohort first, then within MSI strata, as tumors with and without MSI represent clinically and pathologically distinct diseases [66]. We constructed a multivariable Cox proportional hazards model for OS that included variables known to be prognostic in CRC, including race, *KRAS* status, *BRAF* status, MSI status, AJCC stage, and receipt of chemotherapy. Only patients with complete data for all these variables were included. The proportional hazards assumption was checked by including time-by-covariate interaction terms for each covariate in the Cox model. There were no significant interactions between time and any of the covariates. We assessed the prognostic significance of *KRAS* within racial strata by first testing for an interaction between race and *KRAS* status in the Cox model, and secondly by conducting separate Kaplan–Meier analyses within racial strata.

For all analyses, a *p*-value of <0.05 was considered to be significant. All tests were two-sided. All analyses were performed using SPSS v.25.0 (IBM Corp., Armonk, NY, USA).

## 5. Conclusions

We have shown that, between four major races, *KRAS* mutation frequency in CRC does not differ significantly, but *BRAF* mutation frequency does differ significantly. Our results raise the possibility that the differences in *KRAS* mutation rates reported from different countries may either be statistically insignificant or, alternatively, be due to variations in environmental/laboratory factors across countries rather than underlying genetic racial differences. On the other hand, our results suggest that true underlying racial genetic differences may account for the discrepant *BRAF* mutation rates reported from various countries. We have also verified that *BRAF* mutations are less frequent among Middle Easterners, which may have implications for Lynch syndrome screening practices in Middle Eastern populations. With respect to survival outcomes, we showed there was no difference is OS between four major races. Finally, we showed that Europeans may represent a genetically heterogenous population with respect to CRC genetics. However, further studies that include patients from an African background and utilize genetic methods to determine ethnicity are required to verify these results.

## Figures and Tables

**Table 1:** Patient clinicopathological features stratified by race.

Clinicopathological Variable	Total N (%)	Race Unknown N	Caucasian N (%)	Middle Eastern N (%)	Asian N (%)	South American N (%)	*p* ^1^
Median age at diagnosis (N = 413)	66		69	63	56.5	70	<0.001
Sex (N = 445)							0.464
Male	250 (56.2)	13	155 (53.8)	27 (55.1)	42 (61.8)	13 (68.4)	
Female	195 (43.8)	8	133 (46.2)	22 (44.9)	26 (38.2)	6 (31.6)	
Differentiation (N = 381)							0.361
Well	12 (3.1)	0	8 (3.3)	2 (4.7)	1 (1.6)	1 (5.9)	
Moderate	280 (73.5)	10	183 (74.4)	34 (79.1)	43 (68.3)	10 (58.8)	
Poor	89 (23.4)	2	55 (22.4)	7 (16.3)	19 (30.2)	6 (35.3)	
AJCC Stage (N = 397)							0.992
1	5 (1.3)	0	4 (1.6)	1 (2.1)	0 (0)	0 (0)	
2	38 (9.6)	0	26 (10.2)	4 (8.3)	7 (11.3)	1 (5.6)	
3	161 (40.6)	4	101 (39.8)	21 (43.8)	27 (43.5)	8 (44.4)	
4	193 (48.6)	11	123 (48.4)	22 (45.8)	28 (45.2)	9 (50)	
Location (N = 390)							0.126
Right	153 (39.2)	4	101 (40.2)	18 (40)	19 (32.2)	11 (64.7)	
Left	237 (60.8)	14	150 (59.8)	27 (60)	40 (67.8)	6 (35.3)	
Chemotherapy ^2^ (N = 338)							0.527
Yes	328 (97.0)	15	207 (96.7)	44 (100)	51 (94.4)	11 (100)	
No	10 (3)	0	7 (3.3)	0 (0)	3 (5.6)	0 (0)	
*KRAS* status (N = 438)							0.343
WT	258 (58.9)	15	166 (58.9)	25 (52.1)	37 (55.2)	15 (75)	
Mutant	180 (41.1)	6	116 (41.1)	23 (47.9)	30 (44.8)	5 (25)	
*BRAF* status (N = 399)							0.025
WT	358 (89.7)	19	224 (86.5)	39 (100)	58 (93.5)	18 (90)	
Mutant	41 (10.3)	0	35 (13.5)	0 (0)	4 (6.5)	2 (10)	
MMR status (N = 388)							0.882
Proficient	303 (89.6)	15	192 (89.7)	35 (89.7)	46 (86.8)	15 (93.8)	
Deficient	35 (10.4)	1	22 (10.3)	4 (10.3)	7 (13.2)	1 (6.3)	

^1^ Race vs. clinicopathological variable. Fisher’s exact test was used for categorical variables. Kruskal–Wallis test was used to compare age at diagnosis. *p*-values were calculated after excluding patients with unknown race. A *p*-value < 0.05 indicates there is a statistically significant association between race and the relevant clinicopathological variable. ^2^ Any chemotherapy received before death or censoring. Percentages are within race. For each clinicopathological variable, the number of patients for whom data were available is indicated.

**Table 2:** Correlations between *KRAS*/*BRAF*/MMR status and clinicopathological variables.

	*KRAS*			*BRAF*			MMR		
	WT N (%)	Mutant N (%)	*p* ^1^	WT N (%)	Mutant N (%)	*p* ^2^	Proficient N (%)	Deficient N (%)	*p* ^3^
Median age at diagnosis	66	67	0.625	66	69	0.087	66	75	0.028
Sex			0.490			0.095			0.150
Male	140 (57.6)	103 (42.4)		205 (91.9)	18 (8.1)		172 (92)	15 (8)	
Female	115 (61.2)	73 (38.8)		146 (86.4)	23 (13.6)		129 (86.6)	20 (13.4)	
Differentiation			0.006			0.034			0.001
Well	7 (58.3)	5 (41.7)		9 (90)	1 (10)		10 (83.3)	2 (16.7)	
Moderate	144 (53.1)	127 (46.9)		222 (92.9)	17 (7.1)		218 (93.6)	15 (6.4)	
Poor	63 (72.4)	24 (27.6)		70 (83.3)	14 (16.7)		58 (78.4)	16 (21.6)	
T-stage			0.168			0.136			0.297
1	2 (25)	6 (75)		4 (66.7)	2 (33.3)		5 (83.3)	1 (16.7)	
2	9 (64.3)	5 (35.7)		10 (90.9)	1 (9.1)		6 (75)	2 (25)	
3	99 (55.9)	78 (44.1)		147 (91.9)	13 (8.1)		148 (90.8)	15 (9.2)	
4	80 (62.5)	48 (37.5)		104 (87.4)	15 (12.6)		109 (90.1)	12 (9.9)	
N-stage			0.162			0.416			0.037
0	36 (53.7)	31 (46.3)		53 (86.9)	8 (13.1)		46 (88.5)	6 (11.5)	
1	78 (54.9)	64 (45.1)		123 (92.5)	10 (7.5)		114 (85.7)	19 (14.3)	
2	74 (65.5)	39 (34.5)		86 (88.7)	11 (11.3)		104 (95.4)	5 (4.6)	
AJCC Stage			0.317			0.235			0.062
1	1 (20)	4 (80)		3 (75)	1 (25)		1 (100)	0 (0)	
2	21 (55.3)	17 (44.7)		31 (86.1)	5 (13.9)		25 (86.2)	4 (13.8)	
3	98 (60.9)	63 (39.1)		126 (91.3)	12 (8.7)		123 (86.6)	19 (13.4)	
4	103 (56.6)	79 (43.4)		158 (92.9)	12 (7.1)		132 (95)	7 (5)	
Location			0.243			0.001			<0.001
Right	82 (54.7)	68 (45.3)		114 (83.8)	22 (16.2)		116 (82.3)	25 (17.7)	
Left	140 (60.9)	90 (39.1)		193 (94.6)	11 (5.4)		172 (95.6)	8 (4.4)	
Vascular Invasion	116 (51.4)	64 (48.6)	0.022	141 (87.6)	20 (12.4)	0.171	161 (90.4)	17 (9.6)	0.842
Perineural Invasion	48 (59.3)	33 (40.7)	1.0	64 (90.1)	7 (9.9)	1.0	75 (98.7)	1 (1.3)	0.002
Circumferential tumor	86 (66.2)	44 (33.8)	0.041	109 (88.6)	14 (11.4)	0.403	110 (88.7)	14 (11.3)	0.535
Tumor-infiltrating lymphocytes	5 (55.6)	4 (44.4)	1.0	7 (77.8)	2 (22.2)	0.232	4 (44.4)	5 (55.6)	0.001
*BRAF* mutation			<0.001			-			<0.001
WT	194 (55.9)	153 (44.1)	-	-	-		249 (93.3)	18 (6.7)	
Mutant	35 (87.5)	5 (12.5)	-	-	-		18 (54.5)	15 (45.5)	
MMR			0.002			<0.001			-
Proficient	164 (56.0)	129 (44.0)		249 (93.3)	18 (6.7)		-	-	
Deficient	29 (82.9)	6 (17.1)		18 (54.5)	15 (45.5)		-	-	

^1^ *KRAS* status vs. clinicopathological variable. ^2^ *BRAF* status vs. clinicopathological variable. ^3^ MMR status vs. clinicopathological variable. ^1,2,3^ Fisher’s exact test was used for categorical variables. Mann–Whitney U test was used to compare age at diagnosis. Percentages are within clinicopathological variable.

**Table 3:** Correlations between race and *KRAS*/*BRAF*/MMR status in the overall cohort.

	*KRAS*			*BRAF*			MMR		
	WT N (%)	Mutant N (%)	*p* ^1^	WT N (%)	Mutant N (%)	*p* ^2^	Proficient N (%)	Deficient N (%)	*p* ^3^
			0.343			0.025			0.882
Caucasian	166 (58.9)	116 (41.1)		224 (86.5)	35 (13.5)		192 (89.7)	22 (10.3)	
Middle Eastern	25 (52.1)	23 (47.9)		39 (100)	0 (0)		35 (89.7)	4 (10.3)	
Asian	37 (55.2)	30 (44.8)		58 (93.5)	4 (6.5)		46 (86.8)	7 (13.2)	
South American	15 (75)	5 (25)		18 (90)	2 (10)		15 (93.8)	1 (6.3)	

^1^ *KRAS* status vs. race. ^2^ *BRAF* status vs. race. ^3^ MMR status vs. race. ^1,2,3^ Fisher’s exact test was used. Percentages are within race.

**Table 4:** Frequency of specific codon 12 and 13 *KRAS* mutations stratified by race.

N (%)	G12D	G12R	G12V	G12A	G12C	G12S	G13D
Caucasian	40 (13.8)	4 (1.4)	41 (14.4)	23 (7.9)	19 (6.6)	15 (5.2)	17 (5.9)
Middle Eastern	8 (16.7)	1 (2.1)	5 (10.4)	3 (6.3)	1 (2.1)	3 (6.3)	5 (10.4)
Asian	11 (16.4)	0 (0)	9 (13.4)	7 (10.4)	6 (9.0)	3 (4.5)	5 (7.5)
South American	1 (5.0)	0 (0)	1 (5)	1 (5.0)	2 (10.0)	1 (5.0)	2 (10.0)
Missing Race	1	0	1	1	0	1	3
*p*-value ^1^	0.61	0.68	0.75	0.86	0.37	0.95	0.46

^1^ Fisher’s exact test. Percentages are within race. Percentages are calculated using the total number of patients in each race.

**Table 5:** Multivariable Cox Model for OS ^1^.

Variable in Cox Model	Hazard Ratio (95% CI)	*p*
Age	1.022 (1.002–1.042)	0.028
Sex		
Male	1 (ref)	
Female	1.098 (0.731–1.650)	0.652
Race		0.775
Caucasian	1 (ref)	
Middle Eastern	1.389 (0.698–2.802)	0.385
Asian	0.928 (0.51–1.69)	0.808
South American	1.191 (0.473–2.998)	0.710
*KRAS* status		
WT	1 (ref)	
Mutant	0.999 (0.654–1.527)	0.996
*BRAF* status		
WT	1 (ref)	
Mutant	2.278 (1.099–4.722)	0.027
MMR status		
Proficient	1 (ref)	
Deficient	0.332 (0.119–0.929)	0.036
AJCC stage		<0.001
4	1 (ref)	
3	0.302 (0.191–0.479)	<0.001
2	0.877 (0.445–1.727)	0.704
1	0.000 ^2^	0.967
Differentiation		0.009
Well	1 (ref)	
Moderate	3.009 (0.715–12.667)	0.133
Poor	5.459 (1.243–23.975)	0.025
Location		
Right	1 (ref)	
Left	0.728 (0.471–1.125)	0.153
Chemotherapy received ^3^		
No	1 (ref)	
Yes	0.281 (0.096–0.819)	0.020

^1^ The Cox model involves 203 patients for whom complete data were available for the variables listed. ^2^ There were too few cases in this subgroup to calculate a meaningful 95% CI. ^3^ Any chemotherapy received before death or censoring.

**Table 6:** Correlations between ethnic groups and *KRAS*/*BRAF*/MMR status.

	*KRAS*			*BRAF*			MMR		
	WT N (%)	Mutant N (%)	*p* ^1^	WT N (%)	Mutant N (%)	*p* ^2^	Proficient N (%)	Deficient N (%)	*p* ^3^
			0.009			0.010			0.566
Anglo-Saxon	110 (64)	62 (36)		130 (81.8)	27 (17.2)		118 (86.8)	18 (13.2)	
Middle Eastern	25 (52.1)	23 (47.9)		39 (100)	0 (0)		35 (89.7)	4 (10.3)	
Asian	37 (55.2)	30 (44.8)		58 (93.5)	4 (6.5)		46 (86.8)	7 (13.2)	
South American	15 (75)	5 (25)		18 (90)	2 (10)		15 (93.7)	1 (6.3)	
Southern European	26 (68.4)	12 (31.6)		33 (89.2)	4 (10.8)		29 (93.5)	2 (6.5)	
Other European	30 (41.7)	42 (58.3)		61 (90.2)	4 (9.8)		45 (95.7)	2 (4.3)	

^1^ *KRAS* status vs. ethnic group. ^2^ *BRAF* status vs. ethnic group. ^3^ MMR status vs. ethnic group. ^1,2,3^ Fisher’s exact test was used. Percentages are within race.

## Data Availability

The data used for this study are available in the Excel File titled Raw Data (Supplementary File S1). The codes used are available in the document titled Code for Excel Data Sheet (Supplementary Table S5).

## Supplementary Materials

The following supporting information can be downloaded at: https://www.mdpi.com/article/10.3390/ijms242417509/s1.
