# Supplementary material for: KRAS and BRAF Mutation Rates and Survival Outcomes in Colorectal Cancer in an Ethnically Diverse Patient Cohort
Source: Int J Mol Sci. 2023 Dec 15;24(24):17509. doi: 10.3390/ijms242417509 (PMC10743527; doi:10.3390/ijms242417509)
Supplement: Supplementary file 1 [file ijms-24-17509-s001.zip › ijms-2753157-supplementary/ijms-2753157-supplementary-figures.pdf]

Supplementary Figure S1: Kaplan-Meier curves for specific KRAS mutations

A. Kaplan-Meier curves comparing OS between Caucasians and Middle Easterners with KRAS G12D mutations

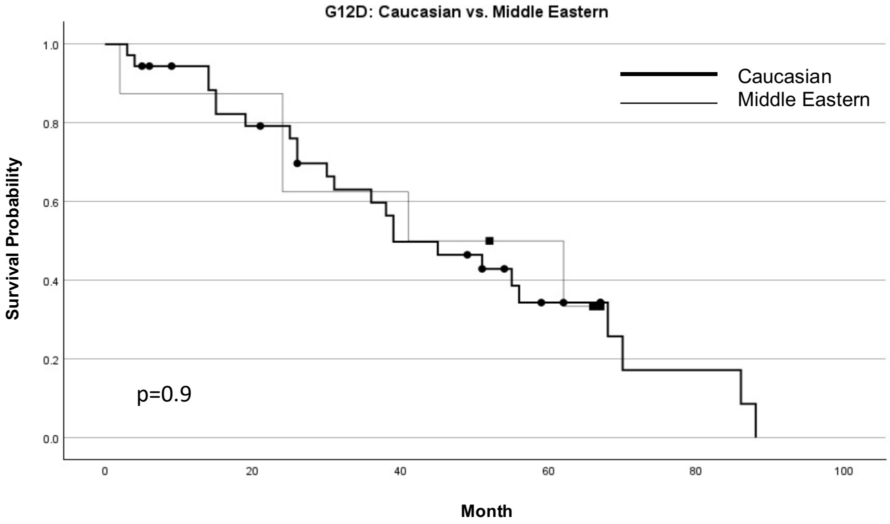

Caucasian: n=36, median OS 39 months, median follow-up 62 months
Middle Eastern: n=8, median OS 41 months, median follow-up 66 months

B. Kaplan-Meier curves comparing OS between Caucasians and Asians with KRAS G12V mutations

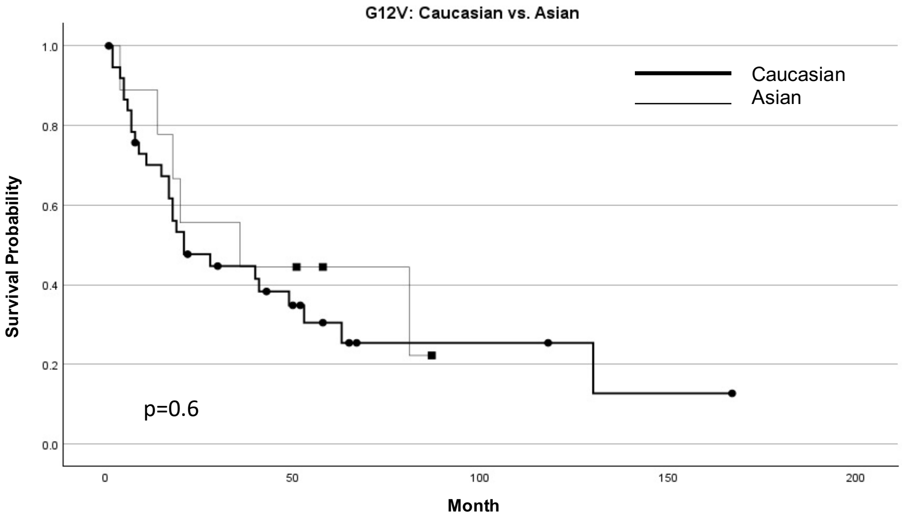

Caucasian: n=38, median OS 21 months, median follow-up 65 months
Asian: n=9, median OS 36 months, median follow-up 58 months

C. Kaplan-Meier curves comparing OS between Caucasians and Asians with KRAS G12A mutations.

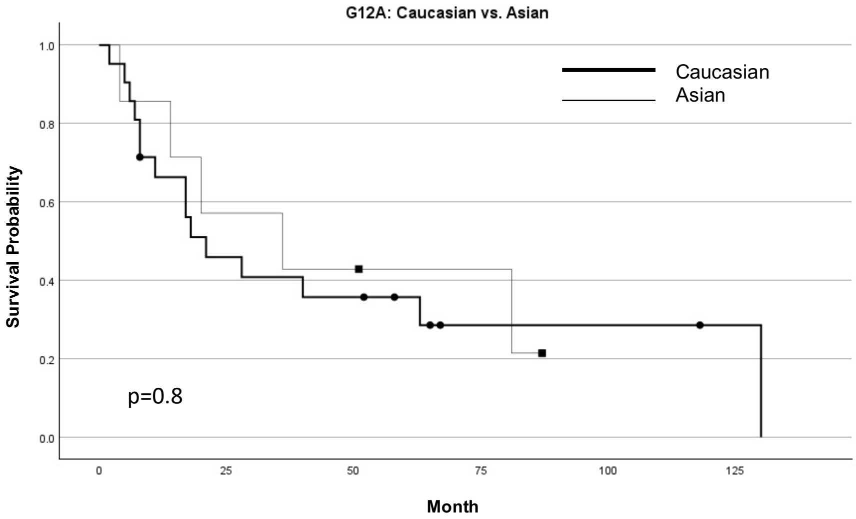

Caucasian: n=21, median OS 21 months, median follow-up 67 months
Asian: n=7, median OS 36 months, median follow-up 87 months

D. Kaplan-Meier curves comparing OS between Caucasians with G12D mutations and Caucasians without G12D mutations

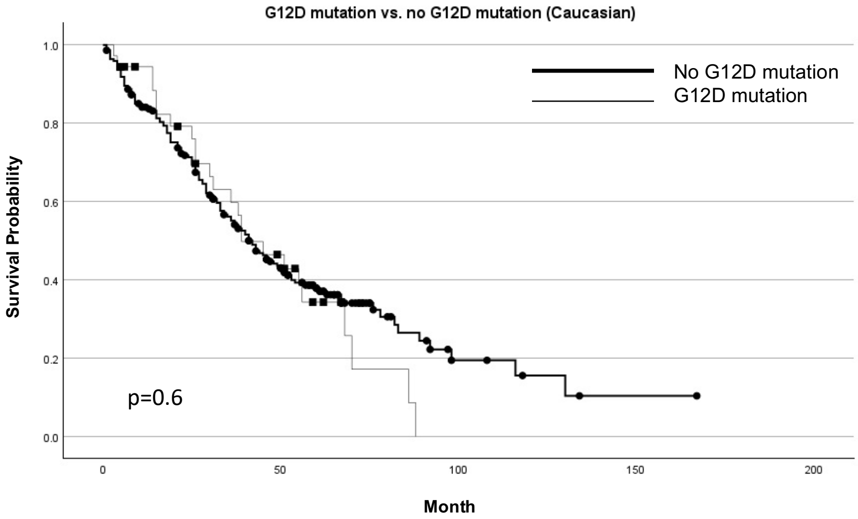

Caucasians with G12D mutation: n=36, median OS 39 months, median follow-up 62 months
Caucasians without G12D mutation: n=222, median OS 42 months, median follow-up 64 months

E. Kaplan-Meier curves comparing OS between Middle Easterners with G12D mutations and Middle Easterners without G12D mutations

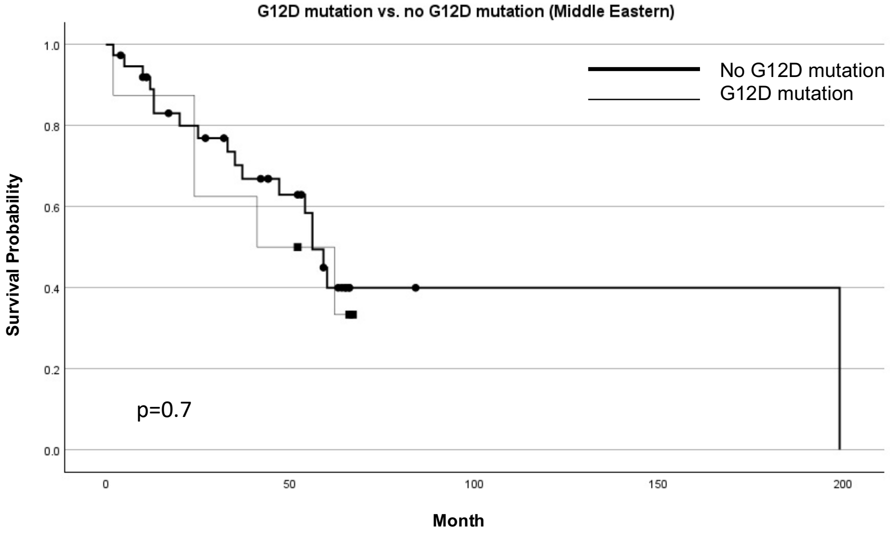

Middle Easterners with G12D mutation: n=8, median OS 41 months, median follow-up 66 months
Middle Easterners without G12D mutation: n=38, median OS 56 months, median follow-up 63 months

Supplementary Figure S2. Kaplan-Meier curves comparing OS between KRAS-WT and KRAS-mutant CRC among stage I-III and stage IV CRC

A. OS: KRAS-WT vs. KRAS-mutant in stage I-III CRC

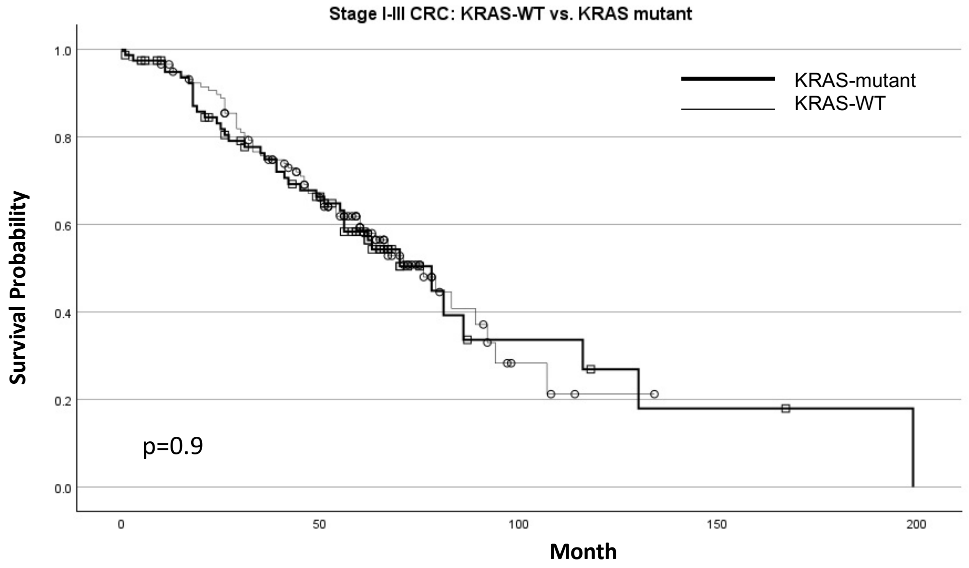

KRAS-WT: n=120, median OS 76 months, median follow-up 66 months
KRAS-mutant: n-82, median OS 78 months, median follow-up 65 months

B. OS: KRAS-WT vs. KRAS-mutant in stage IV CRC

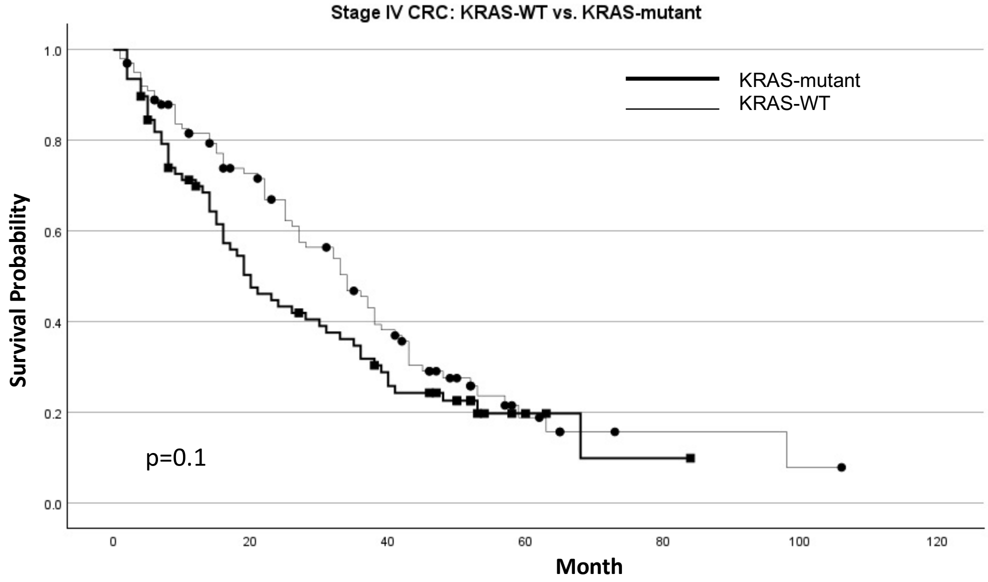

KRAS-WT: n=101, median OS 34 months, median follow-up 52 months
KRAS-mutant: n=78, median OS 20 months, median follow-up 52 months

C. OS: KRAS-WT vs. KRAS-mutant in Caucasians with stage IV CRC

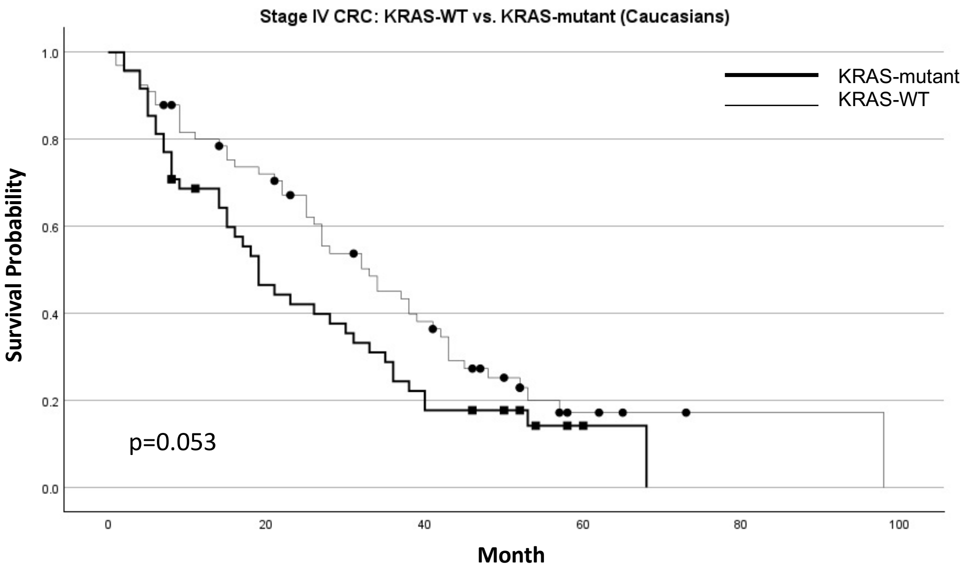

KRAS-WT: n=66, median OS 33 months, median follow-up 57 months
KRAS-mutant: n=48, median OS 19 months, median follow-up 54 months
