# Supplementary material for: KRAS and BRAF Mutation Rates and Survival Outcomes in Colorectal Cancer in an Ethnically Diverse Patient Cohort
Source: Int J Mol Sci. 2023 Dec 15;24(24):17509. doi: 10.3390/ijms242417509 (PMC10743527; doi:10.3390/ijms242417509)
Supplement: Supplementary file 1 [file ijms-24-17509-s001.zip › ijms-2753157-supplementary/ijms-2753157-supplementary-tables.pdf]

Supplementary Table S1: Correlations between race and *KRAS/BRAF* mutation status stratified by MMR status

		KRAS			***BRAF***		
		WT N(%)	Mutant N(%)	P^a	WT N(%)	Mutant N(%)	P^b
**MMR-Proficient**				0.705			0.286
	Caucasian	105 (56.8)	80 (43.2)		155 (91.7)	14 (8.3)	
	Middle Eastern	17 (51.5)	16 (48.5)		28 (100)	0 (0)	
	Asian	23 (51.9)	22 (48.9)		39 (95.1)	2 (4.9)	
	South American	10 (66.7)	5 (33.3)		13 (86.7)	2 (13.3)	
**MMR-deficient**				0.175			0.003
	Caucasian	20 (90.9)	2 (9.1)		6 (30)	14 (70)	
	Middle Eastern	3 (75)	1 (25)		4 (100)	0 (0)	
	Asian	4 (57.1)	3 (42.9)		6 (85.7)	1 (14.3)	
	South American	1 (100)	0 (0)		1 (100)	0 (0)	

[a]*KRAS* status vs. race. [b]*BRAF* status vs. race. [a,b]Fisher's exact test was used.
Percentages are within race and MMR group.

Supplementary Table S2: Correlations between race and *KRAS/BRAF* mutation status stratified by location

		KRAS			***BRAF***		
		WT N(%)	Mutant N(%)	P^a	WT N(%)	Mutant N(%)	P^b
**Right Colon**				0.058			0.154
	Caucasian	61 (61.6)	38 (38.4)		72 (79.1)	19 (20.9)	
	Middle Eastern	7 (41.2)	10 (58.8)		14 (100)	0 (0)	
	Asian	6 (31.6)	13 (68.4)		15 (93.7)	1 (6.3)	
	South American	7 (63.6)	4 (36.4)		9 (81.8)	2 (18.2)	
**Left Colon**				0.394			0.684
	Caucasian	81 (56.2)	63 (43.8)		121 (93.8)	8 (6.2)	
	Middle Eastern	16 (61.5)	10 (38.5)		20 (100)	0 (0)	
	Asian	27 (67.5)	13 (32.5)		34 (91.9)	3 (8.1)	
	South American	5 (83.3)	1 (16.7)		6 (100)	0 (0)	

[a]*KRAS* status vs. race. [b]*BRAF* status vs. race. [a,b]Fisher's exact test was used.
Percentages are within race and location.

Supplementary Table S3: Frequency of specific codon 12 and 13 mutations

***KRAS* mutation**	**G12D**	**G12R**	**G12V**	**G12A**	**G12C**	**G12S**	**G13D**
Frequency	61	5	57	35	28	23	32
Percentage of mutant tumours harbouring mutation (/174 mutated tumours)	35.1	2.9	32.8	20.1	16.1	13.2	18.4

Supplementary Table S4: Details of the Cox Proportional Hazards Model for OS

	B	SE	Wald	df	Sig.	Exp(B)	95.0% CI for Exp(B) Lower	Upper
Sex (*ref.* male)	.094	.208	.203	1	.652	1.098	.731	1.650
Age at Diagnosis	.022	.010	4.850	1	.028	1.022	1.002	1.042
Ethnicity (*ref.* Caucasian)			1.108	3	.775			
Ethnicity (1) (Middle Eastern)	.329	.358	.843	1	.358	1.389	.689	2.802
Ethnicity (2) (Asian)	-.074	.306	.059	1	.808	.928	.510	1.690
Ethnicity (3) (South American)	.175	.471	.138	1	.710	1.191	.473	2.998
KRAS status (*ref.* WT)	-.001	.216	.000	1	.996	.999	.654	1.527
BRAF status (*ref.* WT)	.823	.372	4.905	1	.027	2.278	1.099	4.722
MMR status (*ref.* proficient)	-1.102	.525	4.415	1	.036	.332	.119	.929
AJCC stage (*ref.* stage IV)			26.862	3	.000			
AJCC stage(1)	-12.133	293.976	.002	1	.967	.000	.000	9.197E+244
AJCC stage(2)	-.131	.346	.144	1	.704	.877	.445	1.727
AJCC stage(3)	-1.196	.234	26.066	1	.000	.302	.191	.479
Differentiation (*ref.* well)			9.383	2	.009			
Differentiation(1) (moderate)	1.102	.733	2.257	1	.133	3.009	.715	12.667
Differentiation(2) (poor)	1.697	.755	5.053	1	.025	5.459	1.243	23.975
Location (*ref.* right)	-.318	.222	2.041	1	.153	.728	.471	1.125
Any chemotherapy received before death? (*ref.* No)	-1.269	.546	5.407	1	.020	.281	.096	.819

Reference categories for each covariate are indicated by '*ref.*__'
N=203 patients
Overall model statistics: Chi-square = 60.335, df=15, -2 Log Likelihood = 945.304, p<0.001

Supplementary Table S5: Code for Excel Data Sheet

Variable	Code
**Sex**	
Male	1
Female	2
**Ethnicity**	
Caucasian	1
Middle Eastern	2
Asian	3
South American	4
Southern European	5
Other European	6
***KRAS* status**	
WT	1
Mutant	2
***BRAF* status**	
WT	1
Mutant	2
**MMR status**	
Proficient	1
Deficient	2
**T-stage**	Code number = T stage
**N-stage**	Code number = N stage
**AJCC stage**	Code number = AJCC stage
**Differentiation**	
Well	1
Moderate	2
Poor	3
**Vascular invasion**	
No	0

Yes	1
**Perineural invasion**	
No	0
Yes	1
**Tumour-infiltrating lymphocytes**	
No	0
Yes	1
**Circumferential**	
Absent	0
Present	1
**Location**	
Right	1
Left	2
**Chemotherapy**	
No	0
Yes	1
**OS event code**	
Event not observed	0
Event observed	1
**DFS event code**	
Event not observed	0
Event observed	1
**G12D, G12R, G12V, G12A, G12C, G12S, G13D mutations**	0=mutation absent<br>1=mutation present
